# Establishment of In Vitro Models by Stress-Induced Premature Senescence for Characterizing the Stromal Vascular Niche in Human Adipose Tissue

**DOI:** 10.3390/life12101459

**Published:** 2022-09-20

**Authors:** Marlene Wahlmueller, Marie-Sophie Narzt, Karin Missfeldt, Verena Arminger, Anna Krasensky, Ingo Lämmermann, Barbara Schaedl, Mario Mairhofer, Susanne Suessner, Susanne Wolbank, Eleni Priglinger

**Affiliations:** 1 Ludwig Boltzmann Institute for Traumatology in Cooperation with the AUVA, 1200 Vienna, Austria; 2 Austrian Cluster for Tissue Regeneration, 1200 Vienna, Austria; 3 MorphoMed GmbH, 1030 Vienna, Austria; 4 Christian Doppler Laboratory for the Biotechnology of Skin Aging, Department of Biotechnology, Institute of Molecular Biotechnology, University of Natural Resources and Life Sciences, 1190 Vienna, Austria; 5 Rockfish Bio AG, 1010 Vienna, Austria; 6 University Clinic of Dentistry, Medical University of Vienna, 1090 Vienna, Austria; 7 Department of Hematology and Internal Oncology, Johannes Kepler University, 4020 Linz, Austria; 8 Austrian Red Cross Blood Transfusion Service for Upper Austria, 4020 Linz, Austria

**Keywords:** senescence, stress-induced premature senescence (SIPS), doxorubicin, adipose tissue, adipose-derived stromal/stem cells (ASCs), stromal vascular fraction (SVF), microtissue

## Abstract

Acting as the largest energy reservoir in the body, adipose tissue is involved in longevity and progression of age-related metabolic dysfunction. Here, cellular senescence plays a central role in the generation of a pro-inflammatory environment and in the evolution of chronic diseases. Within the complexity of a tissue, identification and targeting of senescent cells is hampered by their heterogeneity. In this study, we generated stress-induced premature senescence 2D and 3D in vitro models for the stromal vascular niche of human adipose tissue. We established treatment conditions for senescence induction using Doxorubicin (Dox), starting from adipose-derived stromal/stem cells (ASCs), which we adapted to freshly isolated microtissue-stromal vascular fraction (MT-SVF), where cells are embedded within their native extracellular matrix. Senescence hallmarks for the established in vitro models were verified on different cellular levels, including morphology, cell cycle arrest, senescence-associated β-galactosidase activity (SA-βgal) and gene expression. Two subsequent exposures with 200 nM Dox for six days were suitable to induce senescence in our in vitro models. We demonstrated induction of senescence in the 2D in vitro models through SA-βgal activity, at the mRNA level (*LMNB1*, *CDK1*, *p21*) and additionally by G2/M phase cell cycle arrest in ASCs. Significant differences in Lamin B1 and p21 protein expression confirmed senescence in our MT-SVF 3D model. MT-SVF 3D cultures were composed of multiple cell types, including CD31, CD34 and CD68 positive cells, while cell death remained unaltered upon senescence induction. As heterogeneity and complexity of adipose tissue senescence is given by multiple cell types, our established senescence models that represent the perivascular niche embedded within its native extracellular matrix are highly relevant for future clinical studies.

## 1. Introduction

Adipose tissue is a complex organ that shows high metabolic and endocrinological activity while acting as the largest energy reservoir in the body [1]. It is involved in the progression of age-related metabolic dysfunctions, which are linked to several clinical pathologies. Senescent cells accumulate in adipose tissue with age [2,3] and chronic inflammatory diseases, such as obesity, induce an early senescence program, which negatively impacts endogenous repair mechanisms [4]. It was also found that obesity-induced senescence drives anxiety and impairs neurogenesis [5]. Adipose tissue senescence is also linked to defective adipogenesis, inflammation and insulin resistance, leading to Type 2 diabetes [6]. Endothelial progeria induces adipose tissue senescence and impairs insulin sensitivity via the senescence-associated secretory phenotype (SASP), which highlights the importance of vasculatures in fat functions [7]. Senescence in adipose tissue can also impair mesenchymal stem cell (MSC) function and, therefore, negatively impacts autologous cell therapies [4]. Adipose tissue comprises multiple cell types, including mature adipocytes, immune cells, endothelial cells (ECs) and stem and progenitor cells that senesce at different levels with aging or in disease [6]. Senescence profiles are reportedly more dependent on the cell origin than on the mode of senescence–stimuli [8], which emphasizes the heterogeneity in (adipose) tissue senescence. Comprising these multiple cell types and given its easy and relatively painless isolation, subcutaneous adipose tissue also represents an abundant source for regenerative cells that have successfully been applied for reconstructive and regenerative purposes in cell therapies in recent years [9]. Alternatively to culture-expanded adipose-derived stromal/stem cells (ASCs), the stromal vascular fraction (SVF) of cells can be directly used without further processing immediately after isolation for intraoperative autologous therapies in a one-step procedure [10] as the SVF niche bears high regenerative potential in terms of perivascular cells [11,12].

Recently, we described a gentle SVF isolation method, resulting in enriched regenerative cells still protected within their native extracellular matrix, the microtissue-SVF (MT-SVF), suitable for clinical autologous one-step procedures. MT-SVF shows future clinical relevance for treatment of musculoskeletal diseases, since it enhances SVF-based approaches by the possibility to place the cells within their matrix onto defect surfaces, providing a native temporal scaffold [12]. The potential of autologous cell-based therapies might be impacted by the presence of senescent cells derived from aged or diseased donors.

First discovered by Hayflick and Moorhead in 1961, senescence is the cellular state of irreversible growth arrest [13]. This cell fate is a response to various stressors, including telomere shortening after extensive proliferation, genotoxic stress, hypoxia, mitochondrial dysfunction or oncogene activation [14]. In addition to these detrimental effects, senescence also has a beneficial impact, e.g., in tumor suppression [15], wound healing [16] or embryogenesis [17]. The heterogeneity in senescent cell populations that is defined by cell type and senescence stimuli makes detection of senescent cells challenging [18]. In addition, due to the lack of a robust and universal marker, senescent cell identification is based on the analysis of a combination of non-exclusive markers at different cellular levels. Common hallmarks of senescence are induction of the tumor suppressor pathways involved in cell cycle arrest, such as p53/p21^CIP1^ (p21) and pRB/p16^INK4a^ (p16) [19], resulting in overexpression of these cyclin-dependent kinase inhibitors. Senescent cells change their morphology and are characterized by an increase in cell and nuclear size, nuclear rearrangements and appearance of vacuoles [20], leading to a flattened and enlarged phenotype in vitro. Lamin B1 (*LMNB1*) is responsible for nuclear stability and loss of this protein as well as of the mRNA was described as a further hallmark of senescence in vitro and in vivo [21]. Furthermore, senescent cells increase lysosomal mass and express an increased senescence-associated beta-galactosidase (SA-βgal) activity, detectable at pH 6, which is a widely used biomarker [22]. Senescent cells acquire a SASP and release molecules that act immunomodulatory and create an inflammatory microenvironment [23].

Being involved in regenerative and physiological processes, adipose-derived cells are highly relevant for studying senescence. So far, the underlying mechanisms of senescence are not completely understood and information on the senescence status of specific tissues in health, disease and age is limited. Identification of senescent cells within tissues is hampered, especially by the heterogeneity in different cell populations and by the lack of a universal marker for senescence. Since different senescent cell types in adipose tissue affect different cellular processes, including adipogenic capacity in progenitors, lipid handling in ECs or insulin resistance or defective lipolysis in adipocytes [6], a suitable in vitro model to study senescence would be of great value. Previous senescence studies were mostly based on subcutaneous fat biopsies [24] or on human adipose tissue explants [25]. Here, adipocytes represent the dominant cell type that has also been reported to play a role in the development of obesity and insulin resistance when senescent [26].

By the formation of 3D constructs utilizing collagen, an age-related reduction in the neovascular potential of the adipose-derived SVF has been described [27]. Within the present study, we provide a model that represents the stromal vascular niche, without the need for further processing, in its natural environment in 3D for studying the heterogeneity in senescence within the complexity of a tissue. Stress-induced premature senescence (SIPS) is commonly triggered by exposure to sub-lethal stress in terms of oxidative stress [28], irradiation [29] or DNA damaging agents. The cytostatic drug and chemotherapeutic agent Doxorubicin (Dox) induces either apoptosis or senescence in a dose- and time-dependent manner [30,31] and is frequently applied for induction of SIPS [32].

In this study, we established, for the first time, a senescent in vitro model in the MT-SVF. To define treatment conditions for senescence induction, we started from ASCs as 2D monoculture and adapted these treatment conditions to MT-SVF explant cell fractions and to the MT-SVF in full 3D. To characterize these models, the suitability of a variety of markers representing the hallmarks of senescence was tested. We propose that MT-SVF treated by Dox represents a perivascular niche in the context of senescence to study the regenerative potential as well as senescence-associated diseases.

## 2. Material and Methods

### 2.1. Experimental Set-Up

Primary human ASCs seeded on tissue culture plates were used as reference to establish senescent MT-SVF models. Initial dose finding and treatment conditions to trigger SIPS by Dox were investigated. ASCs were exposed to either two (‘2× SIPS’) or three (‘3× SIPS’) subsequent cycles of Dox at different test concentrations (200 nM, 300 nM, 400 nM). The appropriate senescence induction protocol for ASCs that was identified as 2× SIPS 200 nM Dox was further verified and applied to the MT-SVF cultures in three different conditions: SVF-explant cultures derived from MT (‘MT-SVF adherent’), MT-SVF remaining in suspension when cultivated in culture plates (‘MT-SVF suspension’) and MT-SVF cultured in full suspension (‘MT-SVF 3D’). All of the four (2D and 3D) senescent models were generated by exposure to the chosen SIPS protocol and hallmarks of senescence were verified on different cellular levels seven days after Dox deprivation.

### 2.2. Enzymatic Isolation and Cultivation of MT-SVF, SVF and ASCs

The use of human adipose tissue was approved by the local ethical board with patient’s consent. Subcutaneous adipose tissue was collected during routine outpatient liposuction procedures under local tumescence anesthesia. The study includes female individuals at the age of 46.0 ± 6.2 and 40.0 ± 8.0 for MT-SVF isolation and for standard SVF isolation, respectively. MT-SVF isolation was performed by adapting the procedure established by Nürnberger et al. [12], by omitting two centrifugation steps. In brief, 200 mL of liposuction material was transferred into two glass bottles and washed with an equal volume of phosphate-buffered saline (PBS; Lonza, Vienna, Austria). PBS was discarded after separation of adipose tissue from blood and tumescence solution. Tissue was digested by addition of an equal volume of NB4 collagenase (Nordmark, Uetersen, Germany) in PBS containing Ca^2+^/Mg^2+^ and 25 mM *N*-2-hydroxyethylpiperazine-*N*-2-ethanesulfonic acid (HEPES; Sigma-Aldrich, Vienna, Austria) resulting in a final concentration of 0.1 U/mL and incubated at 37 °C for 1 h in a shaking incubator (180 rpm). MT-SVF was collected with a tweezer after digestion and transferred into a cell culture dish (Cellstar; Greiner, Kremsmünster, Austria) followed by washing with PBS and further processing in endothelial growth medium-2 (EGM-2; Lonza). For standard SVF isolation as described by Nürnberger et al. [12], the enzymatically digested tissue was transferred into 50 mL tubes (Greiner) followed by centrifugation for 7 min at 1200× *g*. To eliminate red blood cells, the cell pellets of two 50 mL tubes were pooled into one tube and incubated with 50 mL erythrocyte lysis buffer (154 mM ammonium chloride (Sigma-Aldrich), 10 mM potassium bicarbonate (Sigma-Aldrich), 0.1 mM ethylenediamine-tetraacetic acid (EDTA; Biochrom, Vienna, Austria) in aqua bidest) for 3 min. After centrifugation for 5 min at 500× *g*, the cell pellet was resuspended in 10 mL PBS and filtered through a 100 µm cell strainer (Greiner) by washing the filter with 40 mL PBS for cell dissociation. The cell suspension was centrifuged for 5 min at 500× *g* and resuspended in EGM-2, prior to cell counting and cultivation. After SVF isolation and ASC passaging, cell yield and viability were quantified by an automated cell counter (NucleoCounter^®^ NC-200™; Chemometec, Allerod, Denmark). For this purpose, directly after SVF isolation, disaggregation of cells was performed by lysis buffer and cell yield and viability were determined following the protocol “Viability and Cell Count—Aggregated Cells Assay” staining the cells with 4′,6-diamidino-2-phenylindole (DAPI). For ASCs, following the protocol Mammalian Cells—Viability and Cell Count using the Via1-Cassette™ where the total and the non-viable cell population are stained, identification was based on acridine orange and DAPI according to the manufacturer’s instruction. To obtain ASCs, SVF cells were cultured at 37 °C, 5% CO_2_ and 95% air humidity in EGM-2 as adherent monolayer on tissue culture plastic as described by Priglinger et al. [33]. Media were changed 24 h after SVF isolation and every 3 to 4 days. When a subconfluent state was reached, cells were detached with 1× trypsin/EDTA (Lonza) at 37 °C.

### 2.3. Senescence Induction

**Figure 1:**
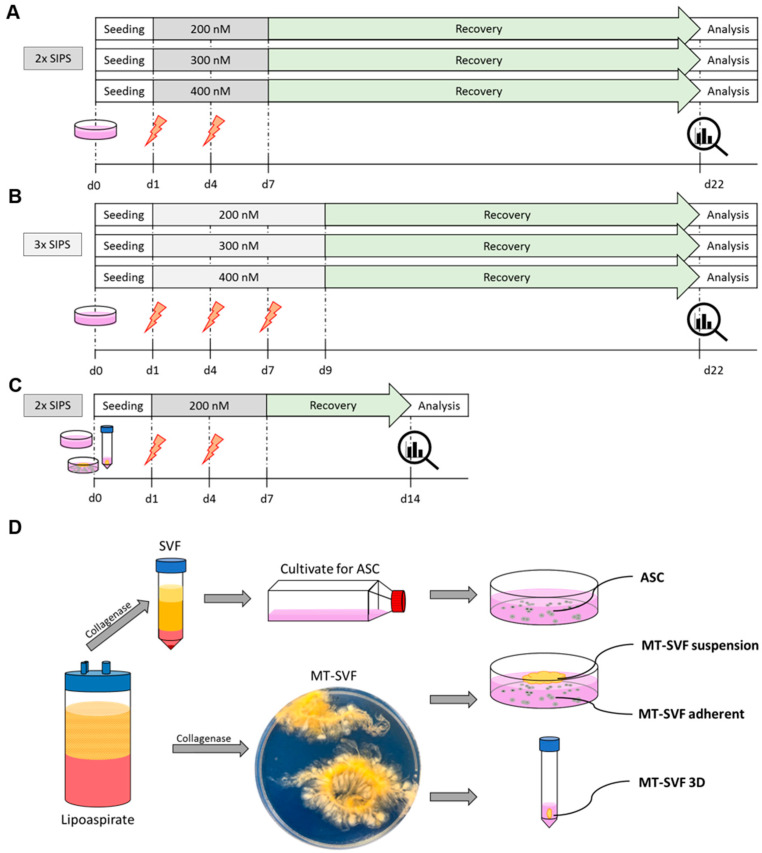
Schematic representation of the experimental set-up for generating senescent in vitro models. A treatment protocol to trigger stress-induced premature senescence (SIPS) by Doxorubicin (Dox) was established with adipose-derived stromal/stem cells (ASCs) of three donors testing different doses and treatment conditions. (**A**,**B**) ASCs were treated on day (d) 1 and d 4 (2× SIPS, (**A**)) and additionally on d 7 (3× SIPS, (**B**)) with 200 nM, 300 nM or 400 nM Dox for 72 h each. Recovery phase was initiated on d 7 (**A**) or on d 9 (**B**). Efficiency of 2× SIPS and 3× SIPS was evaluated by morphological changes and by cumulative population doublings on d 22. (**C**) 2× SIPS with 200 nM Dox was defined as the most efficient SIPS protocol. (**D**) The established treatment protocol was applied to ASCs and to microtissue–stromal vascular fraction (MT-SVF) generating the senescent models MT-SVF suspension, MT-SVF adherent and MT-SVF 3D.

For initial dose finding, senescence induction was adapted from Narzt et al. [34]. In brief, ASCs were seeded into cell culture plates (6 well; Sarstedt, Wr. Neudorf, Austria) at a density of 7400 cells per cm^2^ and either exposed to two (2× SIPS; Figure 1A) or three (3× SIPS; Figure 1B) subsequent Dox doses at different test concentrations. For this purpose, media were changed after 24 h on day (d) 1 and on d 4 for 2× SIPS and additionally on d 7 for 3× SIPS to 2 mL EGM-2 containing 200 nM, 300 nM or 400 nM Dox (Sigma-Aldrich). Recovery phase was initiated on d 7 for 2× SIPS and on d 9 for 3× SIPS by changing to EGM-2 without Dox. During recovery phase media were changed every 3 days. Control cells were treated in the same way, without addition of Dox. Analysis of senescence markers was carried out on d 22. For generation of senescent MT-SVF models, 250–350 mg of freshly isolated MT-SVF was transferred into tubes (15 mL tubes, Greiner) for characterizing the total MT-SVF (MT-SVF 3D) or seeded in cell culture plates (6 well, Sarstedt) for characterizing MT-SVF suspension and the outgrowth MT-SVF adherent culture thereof, in 3 mL EGM-2, respectively, and treated using the 2× SIPS protocol. Control groups were treated in the same way, without addition of Dox. As the reference experiment revealed a stable senescent state induced in ASCs, the phase of Dox deprival was reduced to seven days. Experiments with SIPS-treated conditions as well as with quiescent control cells and with untreated MT-SVF were carried out on d 14, whereas separately cultivated proliferating control cells were analyzed on d 3 for ASC and on d 7 or d 9 for MT-SVF adherent depending on the confluence state.

### 2.4. Morphology and Cumulative Population Doubling (PD)

Images of ASCs were acquired every three days in the same position by a light microscope (Axiovert 200; Zeiss, Jena, Germany) in 10× magnification, for characterizing morphological changes, or in 5× magnification for an overview to evaluate the proliferative capacity. Cells were counted manually and *PD* was calculated by Formula 1, where *N*_0_ is the initial cell number and *N* the final cell number. Lifespan was determined by plotting the cell number per image section against the respective day of analysis. For control cells no lifespan was evaluated due to the high confluence state.

$$PD=\frac{log\frac{N}{N_{0}}}{log2}$$

### 2.5. Cell Cycle Assay

Cell cycle was analyzed by the NucleoCounter^®^ NC-3000™ (Chemometec) following the protocol 2-step cell cycle assay based on DAPI to measure DNA content according to the manufacturer´s instruction. Cells were treated by the SIPS protocol without prior cell cycle synchronization. Briefly, cells were harvested and the pellet was washed with PBS and resuspended in Lysis buffer (Solution 10, Chemometec) supplemented with 10 µg/mL DAPI (Solution 12, Chemometec). The procedure was adapted to the limited amount of cells in the different conditions. Cells were incubated at 37 °C for 5 min before adding an equal volume of Stabilization buffer (Solution 11, Chemometec) and measured in an NC-Slide A2 (Chemometec). DNA content histograms were displayed in the NucleoView™ software and percentages of cells in the different cell cycle stages were quantified.

### 2.6. SA-βgal Activity

Activity of the SA-βgal was analyzed by cytochemical staining modified from Dimri et al. [22], using Senescence β-galactosidase staining kit (Cell Signaling Technology, Leiden, The Netherlands) according to the manufacturer´s instruction. Cytochemical staining was analyzed by manual counting of X-gal-positive cells of random image sections per well. Images were taken on a light microscope (TE2000-S, Nikon, Vienna, Austria). Automated analysis of SA-βgal activity was carried out using the NucleoCounter^®^ NC-3000™ (Chemometec) following the protocol FlexiCyte^TM^. Cells were stained with 33 µM C_12_FDG (Setareh Biotech, LLC, Eugene, OR, USA) in EGM-2 for 2.5 h at 37 °C, 5% CO_2_ adapted from Debacq-Chainiaux et al. [35]. After staining, cells were washed with PBS and trypsinized. The cell pellet was resuspended in PBS supplemented with 10 µg/mL Hoechst-33342 (Solution 15, Chemometec). The procedure was adapted to the limited cell numbers of the different conditions for MT-SVF adherent. Incubation at 37 °C for 15 min followed, prior to loading in an NC-Slide A2. Fluorescence intensities were displayed in the NucleoView™ software as scatter plots and histograms and were further analyzed in the Plot Manager for defining the C_12_FDG-positive cell populations.

### 2.7. RNA Isolation and Quantitative Real-Time PCR (qRT-PCR)

The adherent cell fractions ASC and MT-SVF adherent as well as MT-SVF suspension and MT-SVF 3D were lysed in TriReagent (Sigma-Aldrich) and tissues were additionally shortly vortexed and homogenized in a Thermomixer (Comfort, Eppendorf, Hamburg, Germany) for 2 h at 13,000 rpm at room temperature before storage at −80 °C until RNA isolation. Total RNA was isolated according to the manufacturer´s protocol (Tri Reagent, Sigma-Aldrich). Concentration and purity of RNA were determined using a Biophotometer (6136 Eppendorf). For cDNA synthesis, 500 ng RNA was transcribed using HighCapacity cDNA Reverse Transcription Kit (Applied Biosystems, Vienna, Austria) on a CFX96 Real-Time PCR Detection System (Bio-Rad Laboratories, Vienna, Austria). mRNA levels of the senescence-associated genes *LMNB1*, *CDK1* (*CDC2*), *CDKN1A* (*p21^CIP1^* (*p21*)) and *CDKN2A* (*p16^INK4a^* (*p16*)) were analyzed using qRT-PCR and normalized to the mRNA expression of the reference gene glyceraldehyde-3-phosphate dehydrogenase (*GAPDH*). Relative fold changes in mRNA expression were determined following the 2^−∆∆Ct^ method, as described by Pfaffl [36] and normalized to quiescent control expression levels for the adherent cell fractions or to the expression levels of the untreated control samples for MT-SVF suspension and MT-SVF 3D. Analysis was performed in technical duplicates using LightCycler^®^ 480 SYBR Green I Master (Roche, Mannheim, Germany) reaction mix as in Narzt et al. [37] on a CFX96 Real-Time PCR Detection System (Bio-Rad Laboratories). Forward and reverse primers are listed in Supplementary Table S1.

### 2.8. Immunofluorescence Staining and Immunohistochemical Caspase-3 Staining of MT-SVF Suspension or MT-SVF 3D

MT-SVF suspension and MT-SVF 3D were fixed with 4% buffered Formaldehyde (Carl Roth, Karlsruhe, Germany) overnight and afterwards rinsed by running tap water for 1 h. After dehydration of the samples in a graded series of Ethanol they were stored at 4 °C until further processing. Samples were then embedded in paraffin and 4 µm thin sections were cut. For immunofluorescence staining, after deparaffinization and rehydration, heat-induced epitope retrieval was performed with preheated Sodium Citrate Buffer (10 mM) pH 6 for 20 min. Then the sections were rinsed with TBS and blocked by a 1:60 dilution of Normal Goat Serum (Vector Laboratories, Newark, CA, USA) and 0.1% Bovine Serum Albumin (Merck KGaA, Darmstadt, Germany) in PBS for 1 h. The primary antibodies recombinant anti-p21 ab109520 (1:100; Abcam, Cambridge, UK), anti-CDKN2A/p16INK4a ab54210 (1:1000; Abcam), anti-Lamin B1 ab16048 (1:250; Abcam), anti-CD31 (1:20; Dako, M0823, Clone JC70A), anti-CD34 ab81289 (1:2500; Abcam) and anti-CD68 ab125212 (1:200; Abcam) in blocking solution were applied overnight at 4 °C. Lamin B1 + p16, p21 + p16, CD31 + p21, CD34 + p16 and CD68 + p16 double stainings and Lamin B1 single stainings were performed. The next day, sections were rinsed with TBS and incubated with the secondary antibodies Goat anti-Rabbit 647 (p21, Lamin B1), A32733 (Thermo Fisher Scientific, Waltham, MA, USA), Goat anti-Rabbit Alexa 488 (Lamin B1, CD34, CD68), A32731 (Thermo Fisher Scientific), Goat anti-Mouse Alexa 488 (CD31), A32723 (Thermo Fisher Scientific), Goat anti-Mouse Alexa 647 (p16), A32728 (Thermo Fisher Scientific), Goat anti-Mouse Alexa 488 (p16, CD31) and A32723 (Thermo Fisher Scientific) for 1 h. All secondary antibodies were diluted 1:1000 in blocking solution. Counterstaining of nuclei was performed for 5 min by DAPI FluoroPure™ grade, D21490, (Thermo Fisher Scientific) diluted 1:1000 in TBS. After rinsing the samples in TBS, they were mounted in Mowiol (Carl Roth, Germany). Images were captured with an inverted Olympus IX73 microscope equipped with an Omicron LED hub for 385 nm, 475 nm, 595 nm and 625 nm excitation and a 100×/1.40 UPlanSApo oil immersion objective. For quantification, cells in random image sections were counted manually in a blinded fashion. The senescence-marker-positive cells were determined as percentage of the total DAPI-positive cell count. For immunohistochemical Caspase-3 staining of MT-SVF 3D, sections were deparaffinized and rehydrated followed by EDTA Buffer (pH 9) treatment and blocking in BloxAll (Vector, SP6000). The primary antibody Cleaved Caspase3 (1:400, Asp175, 9661, Cell Signaling Technology) was applied overnight. After rinsing the sections in TBS they were incubated with the secondary antibody anti-Rabbit (BrightVision poly HRP) for 30 min. For detection Nova Red (Vector SK-4805) was used. For quantification of the immunohistochemical staining, cells in random image sections were counted manually in a blinded fashion. The Caspase-3-positive cells were determined as percentage of the total cell count. Images were taken on a light microscope (TE2000-S, Nikon, Vienna, Austria).

### 2.9. Statistics

Statistical analysis was performed using GraphPad Prism 9.2.0 (GraphPad Software, San Diego, CA, USA). Gaussian distribution was tested by Shapiro–Wilk test. In case of Gaussian-distributed data, unpaired t-test (two-tailed) and for non-Gaussian-distribution Mann–Whitney test was performed for following analyses: senescence-associated mRNA expression, SA-βgal activity, Caspase-3 and senescence-associated protein expression. One-way ANOVA with Tukey´s correction for multiple comparisons was used for cell cycle analysis. Statistical significance was set at *p* < 0.05 (* *p* < 0.05, ** *p* < 0.01, *** *p* < 0.001 and **** *p* < 0.0001).

## 3. Results

### 3.1. Reference Experiments in ASCs Identified Most Efficient SIPS Treatment Protocol 

Analysis of cellular morphology and proliferative capacity after 22 days of ASC cultivation identified two subsequent Dox treatments at a concentration of 200 nM for a total treatment period of six days as most effective SIPS treatment protocol (further referred to as ‘SIPS protocol’), with which proliferation and induction of increased cell death were prevented. 

**Figure 2:**
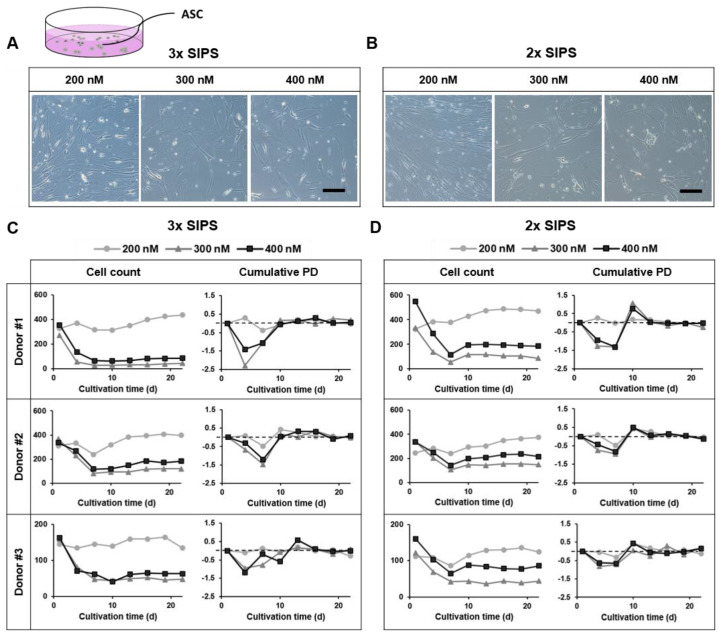
Dose finding and treatment conditions for stress-induced premature senescence (SIPS) in adipose-derived stromal/stem cells (ASCs). (**A**) 3× SIPS at 200 nM, 300 nM or 400 nM induced a senescent phenotype in most cells, whereas some showed long extensions. Scale bar 100 µm. (**B**) 2× SIPS at 300 nM and 400 nM induced a senescent phenotype, additionally treatment at 200 nM generated a heterogeneous population of cells with a senescent or stressed morphology. Scale bar 100 µm. (**C**) 3× SIPS at 300 nM or 400 nM Dox-induced cell death. 2× SIPS at 200 nM maintained cell number without cumulative PD for the first 10 days, except for donor 2, where cell number dropped upon the second Dox administration. (**D**) 2× SIPS by 300 nM and 400 nM Dox lead to a decrease in cell number during the treatment phase and absence in cumulative PD. 2× SIPS by 200 nM maintained cell number and absence of cumulative PD during the treatment phase. In recovery phase cell number of each donor slightly increased and cumulative PD showed an upwards trend. In the further observation period, cell number was mainly constant without cumulative PD.

**Figure 3:**
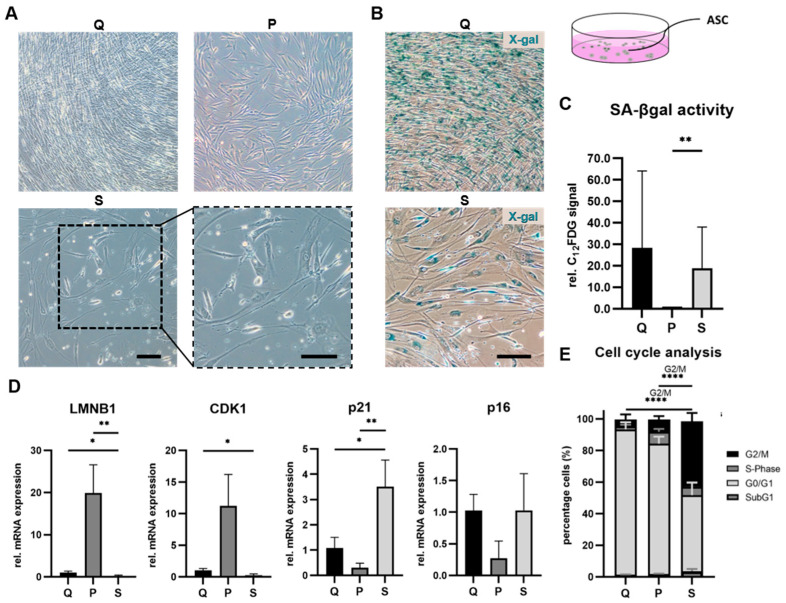
Treatment efficiency of stress-induced premature senescence (SIPS) protocol to trigger senescence in 2D cultured primary adipose-derived stromal/stem cells (ASCs). (**A**) Morphological representative images of quiescent control (Q) ASCs and proliferating control (P) ASCs showed spindle-shaped morphology and 100% confluence, whereas SIPS (S) ASCs revealed a typical senescent or stressed spindle-shaped phenotype (enlarged image section). Scale bar 100 µm. (**B**) Representative images of X-gal staining showed a signal in Q ASCs whereas S ASCs represent senescent cells positive for X-gal. Scale bar 100 µm. (**C**) Quantitative senescence-associated beta-galactosidase activity analyzed for Q, P and S ASCs revealed a significant increase in C_12_FDG-positive cells of S ASCs compared to P ASCs. Data are shown as fold change normalized to P ASCs. (**D**) Gene expression analysis of senescence-associated markers at the mRNA level relative to glyceraldehyde-3-phosphate dehydrogenase (*GAPDH*). S ASCs showed a significant decrease in gene expression of Lamin B1 (*LMNB1*) compared to Q and P ASCs and of Cyclin-dependent kinase 1 (*CDK1*) compared to Q ASCs. A significant increase in cyclin-dependent kinase inhibitor *p21* gene expression of S ASCs compared to Q and P ASCs was observed. (**E**) Cell cycle statistical analysis was only performed for cells in the G2/M phase demonstrating a significant increase in S ASCs compared to Q and P ASCs. Data represent the average of three donors and are shown as fold change normalized to either Q ASCs gene expression or P ASCs or as mean ± standard deviation. Significance levels were denoted as: * *p* < 0.05, ** *p* < 0.01 and **** *p* < 0.0001.

Dox administrations at 200 nM induced a senescent phenotype in the majority of cells of three donors, indicated by an enlarged, flattened morphology and appearance of vacuoles (Figure 2A,B left images) compared to control ASCs that are characterized by a spindle-shaped phenotype (Figure 3A upper images). Regarding 3× SIPS, cell number remained almost constant showing no cumulative PD for the first 10 days, except for donor 2 (cell number dropped from d 4 to d 7 upon the second Dox administration). In 2× SIPS, cell number was maintained and showed no proliferation (no cumulative PD during the treatment phase from d 1 to d 7). During recovery phase, slight proliferation of cells that did not enter senescence was observed in 3× SIPS and 2× SIPS. Most of the surviving cells had entered the senescent state, as no cumulative PD was observed and cell number remained mostly constant (Figure 2C,D).

Dox administrations at 300 nM and 400 nM induced a senescent phenotype in ASCs (Figure 2A,B middle and right images); however, the treatment also induced cell death that is indicated by a decrease in cell number (Figure 2C,D).

### 3.2. Multi-Level Analysis of SIPS ASC Model

To confirm the efficiency of the chosen SIPS protocol for senescence induction in ASCs, a detailed analysis, including morphological changes, SA-βgal activity, cell cycle arrest, as well as senescence-associated mRNA expression, was performed.

#### 3.2.1. SIPS Protocol Triggered Senescent Phenotype in ASCs

SIPS treatment generated a heterogeneous population of ASCs, with the majority of cells developing a typical senescent phenotype. Additionally, some cells showed a stressed morphology and even a spindle-shaped phenotype was observed. In comparison, quiescent control ASCs that are 100% confluent and proliferating control ASCs only showed spindle-shaped morphologies (Figure 3A).

#### 3.2.2. SIPS Protocol Enhanced SA-βgal Activity in ASCs

To confirm the senescent state of ASCs, we further analyzed the activity of SA-βgal, a widely used biomarker of cellular senescence. For qualitative analysis, we performed a cytochemical assay using the substrate X-gal as a first method of detection. SIPS-treated ASCs that acquired a senescent morphology were X-gal positive, identified by blue staining (Figure 3B). We observed that SA-βgal activity was also expressed in quiescent control cells, resulting in a staining of these cells in transient cell cycle arrest (Figure 3B upper image), which has also been described by others [38,39]. Therefore, proliferating cells were identified as the appropriate control for the quantitative fluorescent-based assay for senescence detection. In the next step, SA-βgal activity was detected by the fluorogenic βgal substrate C_12_FDG, where we observed a significant increase in the fluorescence signal of SIPS-treated cells relative to the proliferating control (*p* = 0.0020), whereas no significant alteration in the fluorescence signal could be observed between SIPS-treated cells and quiescent cells (*p* = 0.9952) (Figure 3C).

#### 3.2.3. SIPS Protocol Induced Senescence-Associated mRNA Expression Profile in ASCs

Furthermore, we analyzed mRNA expression of *LMNB1*, a cell-cycle-independent gene as well as of cell-cycle-dependent genes *CDK1* (*CDC2*), *CDKN1A (p21)* and *CDKN2A* (*p16*). Relative mRNA expression was quantified using qRT-PCR. Fold change in gene expression was normalized to the quiescent control, as cells in this condition show similarities to non-dividing senescent cells [40]. We observed a significant downregulation of *LMNB1* mRNA, a major structural component of the nucleus whose loss characterizes cellular senescence, relative to untreated control cells (quiescent; *p* = 0.0192, proliferating; *p* = 0.0071). Moreover, we analyzed gene expression of the cyclin-dependent kinase 1 (*CDK1*), which is involved in cell cycle progression. Here, we observed a significant downregulation of *CDK1* mRNA (*p* = 0.0206) in SIPS-treated ASCs compared to the quiescent control (Figure 3D), which promotes a cell cycle exit at a decision point in the G2 phase (Figure 3E) [41], whereas no significant difference could be observed between SIPS-treated ASCs and proliferating control cells (*p* = 0.1000). Next, we analyzed mRNA expression of the cell-cycle inhibitors *p21* and *p16*. We observed a significant upregulation of *p21* mRNA relative to the quiescent control (*p* = 0.0206) and to the proliferating control (*p* = 0.0065). For *p16*, no significant difference was observed between SIPS-treated ASCs and the untreated control cells (quiescent, *p* = 0.9953; proliferating, *p* = 0.1113) (Figure 3D).

#### 3.2.4. SIPS Protocol Induced Cell Cycle Exit in G2/M Phase in ASCs

Dox-induced senescence was previously reported to arrest cells in G1 and G2/M phase, which is a further hallmark of cellular senescence [42,43]. Cell cycle stages of ASCs were determined without prior cell cycle synchronization. We observed a significant increase in G2/M phase of SIPS-treated ASCs and a contrastingly decreased G0/G1 phase compared to quiescent control (*p ≤* 0.0001) and proliferating control ASCs (*p ≤* 0.0001). No significant difference in cells in the G2/M phase between the control conditions (*p* = 0.5838) was observed (Figure 3E).

### 3.3. Multilevel Analysis of SIPS MT-SVF Adherent Model

To confirm the efficiency of the chosen SIPS protocol for the adherent MT-SVF outgrowth culture, proliferative capacity, morphological changes, SA-βgal activity as well as senescence-associated mRNA expression were analyzed. 

#### 3.3.1. SIPS Protocol Reduced Proliferative Capacity and Triggered Senescent Phenotype in MT-SVF-Adherent Model

**Figure 4:**
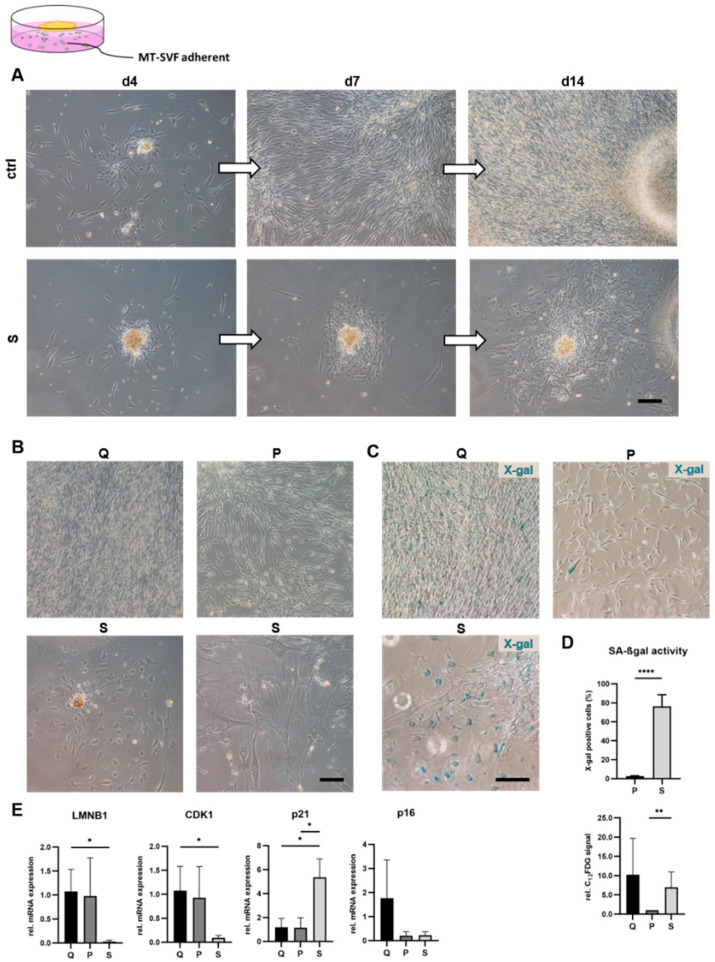
Verification of the stress-induced premature senescence (SIPS) protocol to trigger senescence in MT-SVF adherent culture. (**A**) Monitoring proliferation by acquisition of images on the same position of control (ctrl) and SIPS (S) MT-SVF-adherent cells on d 4, d 7 and d 14. S MT-SVF-adherent cells revealed a lower proliferation potential and a senescent morphology of adipose-derived stromal/stem cells (ASCs) and endothelial cells (ECs) compared to the ctrl. Scale bar 100 µm. (**B**) Morphological representative images of proliferating control (P) and quiescent control (Q) showed a characteristic spindle-shaped morphology of ASCs with 100% confluence in Q and islet formation in P. S MT-SVF-adherent cells showed a typical senescent phenotype of ASCs and ECs on the day of analysis. Scale bar 100 µm. (**C**) Representative images of X-gal staining showed a signal in Q. Phenotypically senescent cells in P and S were positive for X-gal. Scale bar 100 µm. (**D**) Quantitative SA-βgal activity analyzed for P and S revealed a significant increase in percent of X-gal-positive cells of S compared to P (upper graph) as well as in C_12_FDG-positive cells, shown as fold change normalized to P (lower graph). (**E**) Gene expression analysis of senescence-associated markers at the mRNA level relative to glyceraldehyde-3-phosphate dehydrogenase (*GAPDH*). S MT-SVF-adherent cells showed a significant decrease in Lamin B1 (*LMNB1*) and Cyclin-dependent kinase 1 (*CDK1*) gene expression relative to Q and a significant increase in cyclin-dependent kinase inhibitor *p21* gene expression compared to Q and P MT-SVF-adherent cells. Data are shown as fold change normalized to either Q gene expression or to P or as mean ± standard deviation. *n* = 3–5. * *p* < 0.05, ** *p* < 0.01 and **** *p* < 0.0001.

To monitor the proliferative capacity of MT-SVF outgrowth cells, which consist mainly of ASCs and ECs, we acquired images of the same position throughout the cultivation period on d 4, d 7 and d 14 (Figure 4A). Control cells revealed a strong proliferative potential and showed 100% confluence of spindle-shaped ASCs on d 14 (Figure 4A,B), whereas SIPS-treated cells exhibit a lower proliferative potential and a senescent morphology of both cells with an ASC and EC morphology (Figure 4A,B).

#### 3.3.2. SIPS Protocol Enhanced SA-βgal Activity in MT-SVF Adherent Model

To confirm the senescent state of MT-SVF-adherent cells, we further analyzed the activity of SA-βgal. As previously detected in ASCs, X-gal staining resulted in a positive staining of almost all quiescent control cells and was, therefore, excluded as control. Proliferating cells served as appropriate control for analyzing SA-βgal activity (Figure 4C). We observed a significant increase (*p* ≤ 0.0001) in the number of X-gal-positive cells of the SIPS-treated MT-SVF adherent model relative to the proliferating control cells. The fluorescent-based assay using C_12_FDG to detect SA-βgal activity demonstrated a significant increase in the signal relative to the proliferating control (*p* = 0.0046), whereas no significant alteration could be observed between SIPS-treated and quiescent cells (*p* = 0.7000) (Figure 4D).

#### 3.3.3. SIPS Protocol Induced Senescence-Associated mRNA Expression Profile in MT-SVF-Adherent Model

To confirm the senescent state of SVF-explant cells induced by the SIPS protocol, we analyzed mRNA expression of the senescence marker genes *LMNB1*, *CDK1*, *p21* and *p16* using qRT-PCR. Fold change in gene expression was normalized to the quiescent control. We observed a significant downregulation of *LMNB1* mRNA (*p* = 0.0171) and *CDK1* mRNA (*p* = 0.0293) in SIPS-treated SVF-explant cells compared to the quiescent control. Additionally, *p21* mRNA was significantly upregulated relative to the quiescent control (*p* = 0.0131) and to the proliferating control (*p* = 0.0137). For *p16*, no significant difference was observed between SIPS-treated and untreated control cells (quiescent, *p* = 0.1668; proliferating, *p* = 0.2067) (Figure 4E).

### 3.4. Multi-Level Analysis of SIPS MT-SVF Suspension and MT-SVF 3D Models

#### 3.4.1. Immunofluorescence Staining Revealed Senescence-Associated Protein Expression in MT-SVF Suspension and MT-SVF 3D Models

**Figure 5:**
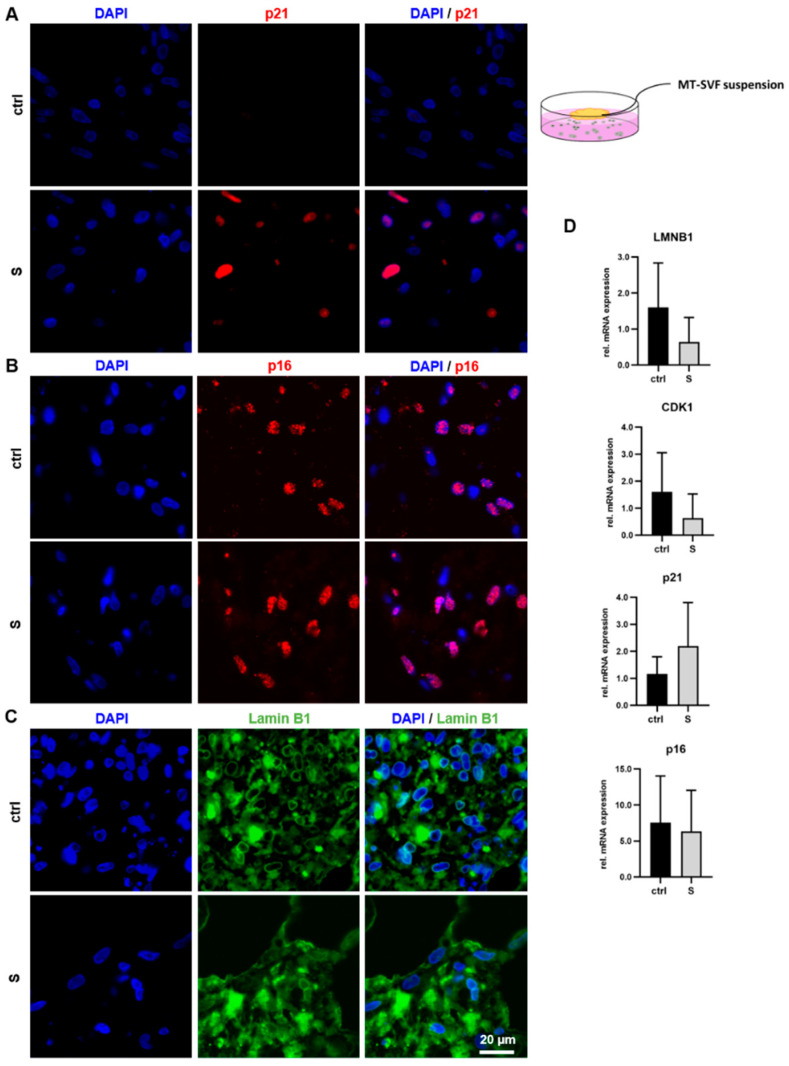
Analysis of stress-induced premature senescence (SIPS) protocol to generate senescent MT-SVF suspension model. (**A**–**C**) Protein expression analysis of p21, p16 and Lamin B1, using immunofluorescence and 4′,6-diamidino-2-phenylindole (DAPI) nuclear staining, was performed for SIPS (S) and untreated control (ctrl) MT-SVF suspension. Scale bar 20 µm. (**A**) p21 was only detected in S MT-SVF suspension and not in ctrl, (**B**) whereas p16 could be also found in the ctrl sample. (**C**) Lamin B1 was observed in the ctrl and in S MT-SVF suspension. A reduction in perinuclear Lamin B1 staining seems to occur in the S MT-SVF suspension. (**D**) Gene expression analysis of senescence-associated markers at the mRNA level relative to glyceraldehyde-3-phosphate dehydrogenase (*GAPDH*). A reduced expression of Lamin B1 (*LMNB1)* and of Cyclin-dependent kinase 1 (*CDK1*) and an increased expression of *p21* was observed in S MT-SVF suspension, although not significant. No difference in *p16* expression was observed between ctrl and S MT-SVF suspension. Data are shown as fold change normalized to untreated ctrl as mean ± standard deviation. *n* = 3.

**Figure 6:**
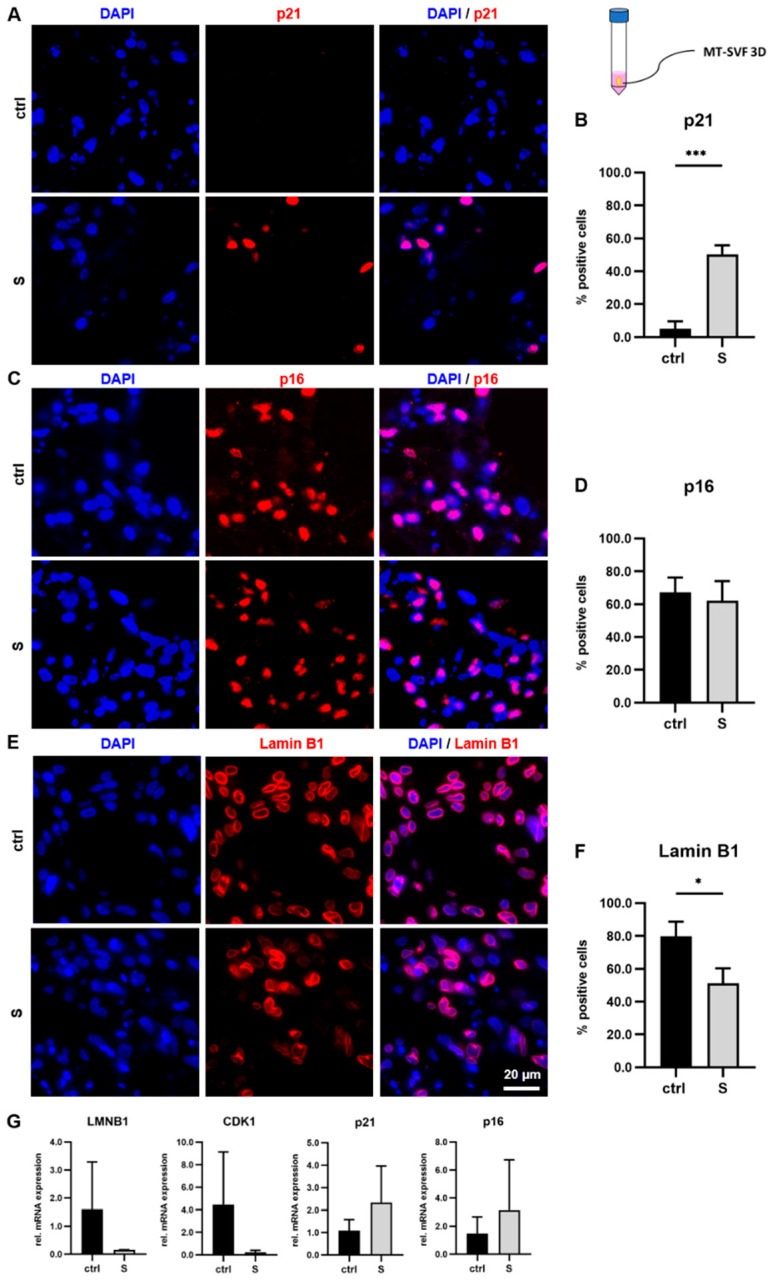
Analysis of stress-induced premature senescence (SIPS) protocol to generate senescent MT-SVF 3D model. (**A**–**F**) Protein expression analysis of p21, p16 and Lamin B1, using immunofluorescence and 4′,6-diamidino-2-phenylindole (DAPI) nuclear staining, was performed for SIPS (S) MT-SVF 3D model and untreated control (ctrl). Scale bar 20 µm. (**A**) p21-positive cells were mainly detected in the S MT-SVF 3D. (**B**) Quantitative analysis revealed a significant increase in percent of p21-positive cells of S MT-SVF 3D compared to the untreated ctrl. (**C**) p16-positive cells were found in the ctrl and in S MT-SVF 3D to the same extent. (**D**) Quantitative analysis revealed no significant difference in percent of p16-positive cells between S MT-SVF 3D and the untreated ctrl. (**E**) Lamin B1 was observed in the ctrl sample and in S MT-SVF 3D. (**F**) Quantitative analysis revealed a significant decrease in percent of Lamin B1-positive cells of S MT-SVF 3D compared to the untreated ctrl. (**G**) Gene expression analysis of senescence-associated markers at the mRNA level relative to glyceraldehyde-3-phosphate dehydrogenase (*GAPDH*). A reduced expression of Lamin B1 (*LMNB1)* and Cyclin-dependent kinase 1 (*CDK1*) and an increased expression of *p21* and *p16* was observed in S MT-SVF 3D, although not significant. Data are shown as fold change normalized to untreated ctrl as mean ± standard deviation. *n* = 3. * *p* < 0.05, *** *p* < 0.001.

We analyzed MT-SVF suspension and MT-SVF 3D models for senescence-associated protein expression using immunofluorescence staining as, with this method, cells in a 3D setting could be visualized. In both models, we observed p21 expression mainly in the SIPS-treated samples (Figure 5A and Figure 6A). The percentage of p21-positive cells in the MT-SVF 3D model was significantly increased (*p* = 0.0004) in the SIPS-treated model compared to the untreated control (Figure 6B). Regarding p16, protein expression was found in the untreated control samples and in both SIPS-treated MT-SVF models (Figure 5B and Figure 6C) with no significant difference (*p* = 0.5820) in the percentage of p16-positive cells (Figure 6D). In addition to being expressed in the untreated control samples, Lamin B1 was observed in both SIPS-treated MT-SVF models (Figure 5C and Figure 6E). Regarding perinuclear Lamin B1 staining, a reduction in SIPS-treated MT-SVF suspension seems to occur (Figure 5C). Percentage of Lamin B1-positive cells in the MT-SVF 3D model was significantly decreased (*p* = 0.0182) in the SIPS-treated model compared to the untreated control (Figure 6F).

#### 3.4.2. mRNA Expression Profile Showed No Senescence-Associated Alteration upon SIPS Treatment in MT-SVF Suspension and MT-SVF 3D Models

We analyzed MT-SVF suspension and MT-SVF 3D for the senescence-associated mRNA expression of *LMNB1*, *CDK1*, *p21* and *p16* after the SIPS treatment. There is a tendency for a downregulation of *LMNB1* and *CDK1* mRNA in MT-SVF suspension (*p* = 0.3002; *p* = 0.3778) (Figure 5D) and MT-SVF 3D (*p* = 0.2103; *p* = 0.1928) (Figure 6G) compared to the untreated control, although without significance. We further observed a tendency for upregulation of *p21* mRNA in both senescent models (MT-SVF suspension: *p* = 0.3595, Figure 5D; MT-SVF 3D: *p* = 0.2749, Figure 6G) and of *p16* mRNA in MT-SVF 3D (*p* = 0.4819) (Figure 6G) without significance. For *p16* in MT-SVF suspension, we did not observe a difference (*p* = 0.8244) between the SIPS-treated sample and untreated control (Figure 5D).

#### 3.4.3. SIPS Treatment Did Not Increase Cell death in MT-SVF 3D 

**Figure 7:**
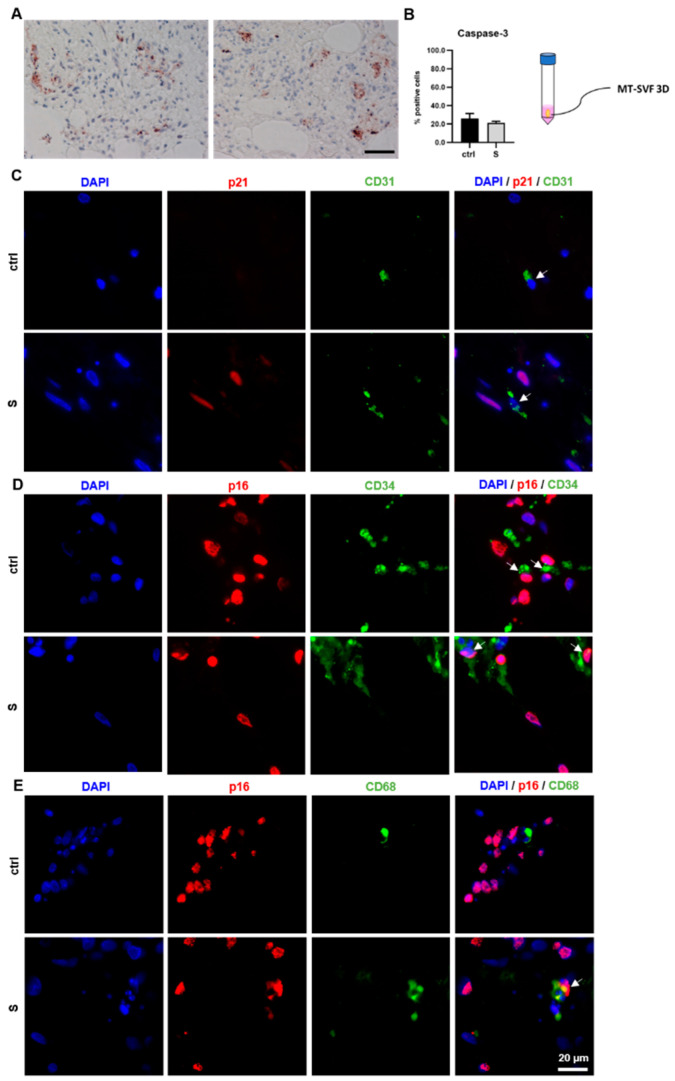
Cell death analysis and cell-type-specific evaluation of stress-induced premature senescence (SIPS) protocol to generate a senescent MT-SVF 3D model. (**A**) Representative images of Caspase-3 immunohistochemical staining showed Caspase-3-positive cells in both, the untreated control (left image) and in SIPS-treated MT-SVF 3D (right image). Scale bar 100 µm. (**B**) Quantitative analysis revealed no significant difference in Caspase-3-positive cells between the untreated control (ctrl) and SIPS-treated (S) MT-SVF 3D. Data are shown as mean ± standard deviation. *n* = 3 (**C**–**E**) Representative cell-type-specific immunofluorescence images of ctrl samples and S MT-SVF 3D, showing nuclear staining by 4′,6-diamidino-2-phenylindole (DAPI) and additional co-staining of senescence markers and cell type markers p21/CD31 (endothelial cells), p16/CD34 (progenitor cells) and p16/CD68 (macrophages). Scale bar 20 µm. (**C**) In S MT-SVF 3D CD31-positive cells (shown by arrow) were not p21 positive. (**D**) In ctrl and S MT-SVF 3D CD34-positive cells co-expressed p16 (shown by arrows) or were p16 negative. (**E**) In ctrl and S MT-SVF 3D few CD68-positive cells were visible, either co-expressing p16 (shown by arrow) or negative for p16.

To exclude increased cell death upon senescence induction by the SIPS treatment protocol, we analyzed the MT-SVF 3D model by immunohistochemical Caspase-3 staining. Here, we could not observe a significant difference (*p* = 0.2126) in Caspase-3-positive cells between SIPS-treated (21.19 ± 1.42%) and untreated control samples (26.03 ± 4.39%), which is, in general, a very low cell death rate in a 3D model after two weeks of cell culture (Figure 7A,B).

#### 3.4.4. Immunofluorescence Co-Expression of Cell-Type-Specific Markers and Senescent Markers in MT-SVF 3D

Immunofluorescence co-staining of cell-type-specific and senescent markers was performed in the MT-SVF 3D model. Cells stained positive for the endothelial marker CD31 were observed in the untreated control and in SIPS-treated samples. SIPS treatment induced p21 expression, whereas co-expression of both markers was not observed (Figure 7C). Representative images show CD34 expression for progenitor cells and high levels of p16 expression in both the untreated control and SIPS-treated sample, identifying CD34/p16-double-positive cells as well as CD34-positive cells without co-expression of the senescence marker (Figure 7D). Regarding the characterized monocyte/macrophage marker, CD68-positive cells were present in the untreated control as well as in the SIPS-treated MT-SVF 3D. In the SIPS-treated sample, CD68-positive cells either co-expressed p16 or were negative for p16 (Figure 7E).

## 4. Discussion

MT-SVF represents a novel and advanced natural scaffold for structural restoration of tissue defects in potential clinical applications. To detect senescent cells and to verify the senescent state of future grafts of diseased or elder donors, we established a SIPS model for adipose-tissue-derived cells in 2D and 3D.

A reference experiment to define treatment conditions for senescence induction in ASCs identified two subsequent exposures by Dox at a concentration of 200 nM for a total treatment period of 6 days as the most efficient treatment protocol. ASCs decreased in number upon exposure to 300 and 400 nM, which confirms the dose-dependent effect of Dox to either induce senescence or apoptosis, described by Lüpertz et al. [30]. The majority of ASCs revealed a senescent phenotype shown by morphological analysis 14 days after Dox deprival. Although some ASCs retained a spindle-shaped and stressed phenotype, no proliferation was observed, which indicates that the majority of ASCs entered the senescent state. As it was reported that Dox-induced senescence shows a time-delayed effect [44], a stable senescent state in the early recovery phase was confirmed by our reference experiment. Based on that finding, we reduced the period of Dox deprival to seven days for further experiments, as described by Narzt et al. [34]. A similar protocol for senescence induction was applied by Kozhukharova et al., who demonstrated that Dox at concentrations ranging from 0.1 to 1 µM irreversibly inhibited cell proliferation for at least four days. They reported that Dox in a low dose induced premature senescence in human MSCs derived from menstrual blood, bone marrow and adipose tissue, whereas high doses of Dox (10–100 µM) induced MSC death within 2–3 days after treatment [45]. In addition to cell proliferation, we analyzed senescence-associated growth arrest, previously described to occur in the G1 and possibly G2 phase of the cell cycle [42]. The later assumption was strengthened by the finding that p21 is involved in G2/M checkpoint regulation [43]. In our study, we observed that Dox treatment induced ASC arrest in the G2/M phase, although cell cycle synchronization was not performed. This is in line with the significant reduction in *CDK1* mRNA expression in ASCs, as this protein plays a key role in cell cycle progression through the G2/M phase [46]. Similar to our study, Dezfouli et al. described lengthening of the G2 phase 14 days after Dox treatment of different aged mouse MSCs [47]. In contrast, others have found that Dox treatment arrested cell proliferation in G1 phase in human ASCs and chondrocytes [32,45]. To the fact that senescent hallmarks are neither exclusively nor universally expressed in senescent cells, we performed a multi-level characterization to confirm the senescent state of our models. A widely used biomarker for detection of senescence is accumulation of lysosomal mass resulting in increased SA-βgal activity that is histochemically identified by X-gal or fluorescent-based using C_12_FDG. As it is postulated that senescent profiles are more dependent on the cell of origin rather than on the mode of induction [8], we could confirm that our established Dox treatment protocol induced accumulation of X-gal and C_12_FDG-positive cells in an ASC culture and in SVF cells outgrown of MT-SVF (MT-SVF adherent). Interestingly, approx. 80% of the outgrowth cells, both ASCs and ECs, were positive for X-gal seven days post Dox treatment, which was verified for cell morphology. Similar results were observed in monocultures by Kozhukharova et al., who identified 90% of X-gal positive ASCs four days post 0.1 µM Dox treatment [45] and by Graziani et al., who measured approx. 80% (values not defined) SA-βgal-positive human umbilical vein endothelial cells (HUVECs) after Dox treatment for 24 h by 250 nM following Dox-free cultivation for eight days [48]. Although p16 is designated as further proof of senescence, we found that *p16* mRNA was not an appropriate marker transcript for senescence detection by qRT-PCR analyses in all of our four senescent in vitro models. In addition to the cell type, senescence markers are also dependent on the level and type of stress by which they are initiated. [49]. Therefore, *p16* mRNA might not be applicable with Dox-induced senescence in this study. Similar results were also described by Casella et al., who detected only a marginal and not significant increase in *p16* mRNA in Dox-induced WI-38 human diploid fibroblast cell senescence [8]. They propose that PURPL, a regulator of p53 levels, is a superior marker to *p16* and *p21* mRNA, confirmed for eight diverse senescence models. Contradictory to that, we could confirm that *p21* mRNA is a suitable marker transcript for senescence detection in our adherent cell models (ASC and MT-SVF adherent). Interestingly, in aged adipose tissue, p16- and p21-positive cells were identified as two distinct cell populations in different tissue locations with different accumulation kinetics [50]. Although our adipose-tissue-derived stromal vascular niche models are composed of multiple cell types, we only observed p21-positive cells in the SIPS-treated MT-SVF and not in the control samples by immunofluorescence. Wang et al. found that p21-positive cells are mainly EC, MSC and myeloid cells, while p16-positive cells are scarce in aged mice visceral fat [50]. We could not confirm p21 in the EC population when co-staining MT-SVF 3D for CD31/p21. Notably, p16-positive cells were observed in both treated models and controls at relatively high levels, without a significant difference, which, in our case, indicates that p16 is not an appropriate marker. This was confirmed by Hall et al., who reported that expression of p16 in macrophages occurs as part of a physiological response to immune stimuli in adipose tissue of old mice [51]. CD68-positive cells (macrophages) were present either co-expressing p16 or without co-expression of this senescence marker. This might contribute to the p16 expression levels in both control samples and SIPS-treated samples of MT-SVF models, as we have previously found that MT-SVF comprises substantial numbers of CD68 (monocyte, macrophage)-positive cells [12]. In addition, we identified p16-positive and p16-negative progenitor cells (CD34-positive) in SIPS-treated samples. The 3D model comprising multiple cell types is, hence, suited to identify senescence at protein level in different cell types comprised in the stromal vascular niche of adipose tissue. Furthermore, we could confirm senescence for the ASC and MT-SVF-adherent models by *LMNB1* loss, a senescence-associated biomarker, irrespective of cell growth arrest and senescence inducer [21]. Within our 3D cultured tissue model, we observed a significant decrease in Lamin B1-positive cells compared to the untreated control on protein level and reduction in *LMNB1* mRNA levels, although not to a significant degree, which might be due to high donor variability. This is similar to other observations where LMNB1 loss was demonstrated on the protein and mRNA level as a consequence of different senescence inducers in different aging mouse tissues [52] and also in human cells and tissues upon UV exposure at transcript and protein levels [53]. Saleh et al. reported LMNB1 loss as therapy-induced senescence marker in breast cancer samples upon incomplete chemotherapy including Dox [54]. The observed heterogeneity in senescence levels originally present within the MT-SVF derived from subcutaneous adipose tissue and the different levels induced by Dox treatment in vitro reflect the previously described variations in senescence within tissues. The MT-SVF in vitro models are, therefore, suited to represent the higher complexity of the senescence status in vivo and, hence, may be appropriate models to study the stromal vascular niche.

## 5. Conclusions

In this study, we established in vitro models, characterized by a comprehensive multi-level analysis of senescence hallmarks, to study senescence in adipose tissue. Starting from human ASCs, we generated in vitro models that represent the SVF niche in adipose tissue. We could model the perivascular niche embedded within its native extracellular matrix, which is highly relevant for future clinical studies in the context of senescence. As multiple cell types lead to a heterogeneity and complexity in adipose tissue senescence, our microtissue models facilitate analysis of the endogenous senescent state. Although senescence of adipose tissue has previously been studied in vitro in 2D expanded ASCs, as well as in 3D explants and biopsies, MT-SVF representing the 3D stromal vascular niche and equally a natural scaffold enriched in regenerative cells should be of great value to study adipose tissue pathologies linked to senescence, such as obesity and metabolic disease.

## Data Availability

The data presented in this study are available from the corresponding author upon reasonable request.

## Supplementary Materials

The following supporting information can be downloaded at www.mdpi.com/article/10.3390/life12101459/s1, Table S1: Primers used for real-time qRT-PCR.
