# Supplementary material for: Establishment of In Vitro Models by Stress-Induced Premature Senescence for Characterizing the Stromal Vascular Niche in Human Adipose Tissue
Source: Life (Basel). 2022 Sep 20;12(10):1459. doi: 10.3390/life12101459 (PMC9605485; doi:10.3390/life12101459)
Supplement: Supplementary file 1 [file life-12-01459-s001.zip › life-1923993-supplementary.pdf]

Table S1. Primers used for real-time qRT-PCR.

Gene Name	Symbol	Sequence 5′–3′
Glyceraldehyde-3-phosphate dehydrogenase	GAPDH	f:CAGTCAGCCGCATCTTCTTTTG<br>r: CGCCCAATACGACCAAATCC
Lamin B1	LNMB1	f: TGGAAGAATCAGAGGCGAGT<br>r: TGCAGCCCAAATTGTAACAG
Cyclin dependent kinase 1	CDK1 (Cdc2)	f: AAGCCGGGATCTACCATACC<br>r: GGAACCCCTTCCTCTTCACT
Cyclin dependent kinase inhibitor 1A	CDKN1A ($p21^{CIP1}$)	f: CAGCAGAGGAAGACCATGTG<br>r: GGCGTTTGGAGTGGTAGAAA
Cyclin dependent kinase inhibitor 2A	CDKN2A ($p16^{INK4a}$)	f:CAACGCACCGAATAGTTACG<br>r: ACCAGCGTGTCCAGGAAG
